# All That Glisters Is Not Gold: Sampling-Process Uncertainty in Disease-Vector Surveys with False-Negative and False-Positive Detections

**DOI:** 10.1371/journal.pntd.0003187

**Published:** 2014-09-18

**Authors:** Fernando Abad-Franch, Carolina Valença-Barbosa, Otília Sarquis, Marli M. Lima

**Affiliations:** 1 Infectious Disease Ecology Laboratory, Instituto Leônidas e Maria Deane – Fiocruz Amazônia, Manaus, Brazil; 2 Chagas Disease Eco-epidemiology Laboratory, Instituto Oswaldo Cruz – Fiocruz, Rio de Janeiro, Brazil; The Faculty of Medicine, The Hebrew University of Jerusalem, Israel

## Abstract

**Background:**

Vector-borne diseases are major public health concerns worldwide. For many of them, vector control is still key to primary prevention, with control actions planned and evaluated using vector occurrence records. Yet vectors can be difficult to detect, and vector occurrence indices will be biased whenever spurious detection/non-detection records arise during surveys. Here, we investigate the process of Chagas disease vector detection, assessing the performance of the surveillance method used in most control programs – active triatomine-bug searches by trained health agents.

**Methodology/Principal Findings:**

Control agents conducted triplicate vector searches in 414 man-made ecotopes of two rural localities. Ecotope-specific ‘detection histories’ (vectors or their traces detected or not in each individual search) were analyzed using ordinary methods that disregard detection failures and multiple detection-state site-occupancy models that accommodate false-negative and false-positive detections. Mean (±SE) vector-search sensitivity was ∼0.283±0.057. Vector-detection odds increased as bug colonies grew denser, and were lower in houses than in most peridomestic structures, particularly woodpiles. False-positive detections (non-vector fecal streaks misidentified as signs of vector presence) occurred with probability ∼0.011±0.008. The model-averaged estimate of infestation (44.5±6.4%) was ∼2.4–3.9 times higher than naïve indices computed assuming perfect detection after single vector searches (11.4–18.8%); about 106–137 infestation foci went undetected during such standard searches.

**Conclusions/Significance:**

We illustrate a relatively straightforward approach to addressing vector detection uncertainty under realistic field survey conditions. Standard vector searches had low sensitivity except in certain singular circumstances. Our findings suggest that many infestation foci may go undetected during routine surveys, especially when vector density is low. Undetected foci can cause control failures and induce bias in entomological indices; this may confound disease risk assessment and mislead program managers into flawed decision making. By helping correct bias in naïve indices, the approach we illustrate has potential to critically strengthen vector-borne disease control-surveillance systems.

## Author Summary

Vector-borne disease prevention often relies on health agents inspecting dwellings and eliminating the vector infestation foci they detect. The effectiveness of prevention programs thus depends on vector-detection performance. Unfortunately, detection failures can be common, particularly when infestation is rare and vector foci small. Although this can threaten vector control, the actual performance of vector searches has seldom been investigated in detail. Here, we assess Chagas disease vector detection by trained control-surveillance agents. We used models that explicitly account for detection errors to analyze triplicate vector detection/non-detection records from 414 man-made ‘ecotopes’ (houses, henhouses, woodpiles, etc.) in two rural localities. On average, a single round of vector searches correctly identified about 28% of the infested ecotopes; detection was more challenging in lightly-infested ecotopes and in some ecotope types, particularly houses and brick piles. After correcting detection errors, we estimated that ∼45% of the ecotopes were most likely infested, while observed rates were ∼11–19%; standard, single-round vector searches therefore missed many infestation foci. Our findings underscore the importance of taking detection failures into account when assessing infestation by disease vectors, and illustrate a straightforward approach to tackle the major but still underappreciated problem of imperfect vector detection.

## Introduction

The primary prevention of most vector-borne diseases depends on averting contact between humans and pathogen vectors [1]. In turn, vector control often relies on the detection and elimination of infestation foci, particularly when the vectors occur in or around human residences. This is the case, for example, of the *Aedes* mosquito vectors of dengue and other arboviruses [1], [2] or of the triatomine bug vectors of *Trypanosoma cruzi*, the agent of Chagas disease – the most important human parasitic disease in the Americas (see refs. [1], [3], [4] and http://www.who.int/mediacentre/factsheets/fs340/en/). Since undetected vector foci usually cannot be eliminated, the effectiveness of vector-detection methods can have a strong influence on our ability to prevent new disease cases. In addition, measures of vector occurrence in or around houses (‘infestation’ and related indices) are among the principal indicators used in disease risk assessment and vector control program management – including intervention design, planning, implementation, operation, and evaluation [1]–[3]. Developing and running sound vector-borne disease prevention programs therefore demands a reliable understanding of the vector-detection process; however, few quantitative studies have fully addressed this issue in realistic field settings.

Particularly critical is knowledge about the sensitivity and specificity of the methods used to determine infestation in control-surveillance systems [5]–[9]. In this context, sensitivity is defined as the probability of detecting at least one vector in a site (e.g., a house or any other discrete ‘ecotope’ such as a corral, henhouse, catch basin, or palm-tree) that is actually infested; more generally, sensitivity is the probability of detection, conditioned on occurrence [5]–[9]. If sensitivity is less than 1.0 (<100%), some sites will be classified as non-infested despite being, in fact, infested – i.e., there will be some *false-negative* results in the record database and infestation indices will be biased low [7], [8]. Specificity is, in turn, the probability of declaring non-infested a sampling unit where the vectors indeed do not occur; that is, the probability of non-detection, conditioned on non-occurrence. If this probability is less than 1.0, some sites will be classified as infested when they are not – i.e., there will be some *false-positive* results in the record database, which will induce positive bias in infestation indices [9]. The probability of obtaining a false-positive result equals 1− specificity. Although false-positive results are unlikely to be common in vector surveys, they may possibly arise because of taxonomic errors (say, a non-triatomine reduviid nymph misidentified as a triatomine bug, or a non-vector sandfly species as a vector species) or, more easily, when indirect signs of infestation are used as proxies of vector presence (e.g., triatomine bug fecal streaks, which may be confused with those of other arthropods [10]–[12]) or when householders' reports of vector presence in dwellings are not confirmed by actually examining the insects (e.g., ref. [13]).

In addition to estimating sensitivity and specificity, researchers and program managers may be interested in knowing how these key parameters vary in response to independent variables. For example, we may wish to know whether and to what extent the sensitivity of a vector-detection method is affected by the characteristics of vector hiding/breeding sites (i.e., ecotope traits), by the awareness of vector control agents, or by differences in vector abundance among ecotopes or over time. This latter possibility is particularly relevant in areas undergoing vector control, because the expected effect of control activities is to reduce infestation prevalence, with foci becoming rarer, and vector population density, with foci becoming smaller. In turn, these effects may be expected to reduce the sensitivity of any vector-detection method: rarer and smaller foci will probably be harder to detect [14]–[17].

Unfortunately, no gold-standard vector-detection method (with 100% sensitivity and 100% specificity) is currently available. In the case of Chagas disease vectors, demolition of houses or other man-made structures could perhaps reach near-perfect performance [14], but this option has little practical relevance; as a rule, more sensitive methods are more costly [5], [16]. In this paper, we adopt a different approach based on repeated-sampling of individual ecotopes and the hierarchical site-occupancy models developed by Miller et al. [9], which explicitly accommodate false-negative and false-positive results. This allows us to investigate the sensitivity and specificity of active triatomine-bug searches by trained staff (the standard method used in routine surveillance) with unprecedented detail. We quantify how vector-search sensitivity varies with observed vector density and across ecotope types while adjusting for possible effects of our sampling scheme. We show that triatomine-search specificity is more than acceptable, but sensitivity is overall low and can vary widely, leading to negatively-biased naïve infestation indices that can seriously threaten vector control program management and, ultimately, disease prevention.

## Methods

### Ethics statement

This study is part of a research program on Chagas disease eco-epidemiology approved by Fiocruz's Institutional Review Board (CEP/Fiocruz protocol 139/01) and Committee for Animal Research (CEUA/Fiocruz protocol P59-12-2) and by the Brazilian Environmental Agency (IBAMA/Sisbio protocol 14323-6). All householders provided informed consent prior to dwelling inspections.

### Study setting

We studied two neighboring areas in the lower Jaguaribe valley (state of Ceará, Brazil), where dwelling infestation by triatomine bugs is common and Chagas disease a significant public health concern [18]–[21]. These areas belong, respectively, to the municipalities of Russas and Jaguaruana; while geographically close and ecologically similar, our study localities have some contrasting characteristics. In Russas (∼4°56′S, 37°55.5′W) we studied a rural area consisting of several dwelling clusters plus some isolated dwelling compounds; this area lies close to the main (paved) road and is 4 km from the municipality's main town. The landscape is heavily anthropogenic, with small agricultural plots and a few patches of Caatinga xeric shrubland. In Jaguaruana (∼4°52′S, 37°52′W), the study area is 8–10 km from the municipality's main town, the original Caatinga vegetation is overall better preserved, and dwelling compounds are more spatially scattered; a detailed description of this area can be found in ref. [21].

### Sampling strategy

Our sampling units were all individual ecotopes within each dwelling compound. An ecotope was defined as any man-made discrete structure where triatomine bugs might find shelter; a typical dwelling compound had about 5–6 such ecotopes (mean 5.75, median 5.5, range 2–12) including the house and several further structures (see Table 1 and below). Overall, 414 ecotopes were sampled in 72 dwelling compounds; a few uncommon ecotopes (three kennels, a dovecot, and a bird-cage), none of which appeared to be infested, were excluded from the analyses.

**Table 1:** Naïve indices of infestation (given as percentages) by Chagas disease vectors in 414 man-made ecotopes of the lower Jaguaribe valley in northeastern Brazil after three vector-search rounds.

Ecotope	*n*	All detections				‘Certain’ detections			
		S1	S2	S3	Combined	S1	S2	S3	Combined
House	72	8.33	6.94	5.56	12.50	5.56	2.78	5.56	9.72
Storeroom	19	21.05	15.79	21.05	26.32	21.05	10.53	10.53	26.32
Henhouse	81	12.35	12.35	9.88	17.28	12.35	9.88	9.88	16.05
Goat/sheep corral	41	39.02	24.39	14.63	41.46	36.59	14.63	14.63	41.46
Cattle corral	9	22.22	11.11	11.11	22.22	22.22	0.00	11.11	22.22
Pigsty	38	18.42	13.16	10.53	21.05	10.53	7.89	10.53	15.79
Brick pile	30	0.00	0.00	3.33	3.33	0.00	0.00	3.33	3.33
Tile pile	68	22.06	20.59	11.76	29.41	16.18	17.65	10.29	23.53
Woodpile	56	32.14	23.21	19.64	37.50	30.36	21.43	19.64	35.71
Total	414	18.84	14.73	11.35	23.43	16.18	10.87	10.63	21.01

*n*, number of ecotopes sampled within each class; ‘All detections’ include the detection of only fecal streaks identified (perhaps incorrectly) as those of triatomine bugs, whereas detections were considered ‘certain’ when at least one triatomine bug or exuvia (molted ‘skin’) were found and identified without doubt; S1 to S3, first to third vector-search rounds; Combined, combined results of all three vector-search rounds (percentage of ecotopes with at least one detection in at least one search round).

Each ecotope was searched three times over a short period (median 8 days, range 7–13 days) by local vector control-surveillance staff, for a total effort of 1,242 individual vector searches. Vector-search teams were rotated and kept blind to the results of previous search rounds so that the outcomes of individual vector searches could be treated as independent. Field teams were instructed to stop searching in each ecotope as soon as the first triatomine bug was detected. All ecotopes were sprayed with a pyrethroid insecticide (following Ceará state's Health Department standard procedures) after the second vector search, regardless of whether or not vectors had been detected previously; the third vector search was conducted immediately after insecticide application, which might reveal cryptic infestation foci because of the irritant and ‘knock-down’ effects of pyrethroids [14], [15]. All triatomines found in each ecotope were collected after the third search round. A more detailed description of our sampling scheme, including caveats, can be found in ref. [21]; one important difference between ref. [21] and our present analyses is that here we consider two types of evidence of ecotope infestation: (i) ‘certain’ evidence, represented by the finding and identification of triatomine bugs of any stage or their exuviae (molted ‘skins’), and (ii) ‘uncertain’ evidence, represented by the finding of *only* fecal streaks identified by field staff as triatomine bug feces – a proxy for triatomine bug presence used in vector surveillance in our study setting and elsewhere (e.g., [10]–[12], [14]–[16]). Triatomine bug fecal streaks are relatively easy to distinguish from, but can still be confused with, those of cockroaches, ticks, flies, or bedbugs; hence, this proxy introduces the possibility of false-positive detections [11], [12].

Individual vector-search results in each ecotope were recorded separately so that a three-entry ‘detection history’ including three ‘detection states’ was available for each ecotope: ‘certain’ detections (coded as 2), ‘uncertain’ detections (coded 1), or ‘non-detections’ (coded 0) [9]. Table 2 presents the interpretation of the ‘detection histories’ observed in our survey.

**Table 2:** Chagas disease vector ‘detection histories’ in 414 man-made ecotopes of the lower Jaguaribe valley in northeastern Brazil across three vector-search rounds: code, interpretation, and individual history frequencies.

Vector-search round			Interpretation	Frequency
Search 1	Search 2	Search 3		
0	0	0	No detection after three search rounds	317
0	0	2	Vectors/exuviae detected only in search 3	7
0	1	0	Only fecal streaks detected only in search 2	3
0	2	0	Vectors/exuviae detected only in search 2	7
0	2	2	Vectors/exuviae detected in searches 2 and 3	2
1	0	0	Only fecal streaks detected only in search 1	5
1	0	2	Only fecal streaks detected in search 1; vectors/exuviae detected in search 3	1
1	1	0	Only fecal streaks detected in searches 1 and 2	2
1	2	0	Only fecal streaks detected in search 1; vectors/exuviae detected in search 2	2
1	2	2	Only fecal streaks detected in search 1; vectors/exuviae detected in searches 2 and 3	1
2	0	0	Vectors/exuviae detected only in search 1	16
2	0	2	Vectors/exuviae detected in searches 1 and 3	7
2	1	0	Only fecal streaks detected in search 2; vectors/exuviae detected in search 1	6
2	1	1	Only fecal streaks detected in searches 2 and 3; vectors/exuviae detected in search 1	1
2	1	2	Only fecal streaks detected in search 2; vectors/exuviae detected in searches 1 and 3	4
2	2	0	Vectors/exuviae detected in searches 1 and 2	9
2	2	1	Only fecal streaks detected in search 3; vectors/exuviae detected in searches 1 and 2	2
2	2	2	Vectors/exuviae detected in searches 1 to 3	22

Results in the first three columns are coded as follows: 0 =  non-detection, 1 =  detection of only fecal streaks suggestive of triatomine bug presence, and 2 =  detection of at least one triatomine bug (any stage) or exuvia (molted ‘skin’) that could be identified without doubt.

### Data analysis

The focus of this paper is the sampling process governing vector detection/non-detection, not the biological processes governing vector presence/absence in individual ecotopes. Therefore, and for simplicity, we pool data across triatomine bug species (*Triatoma brasiliensis*, *T. pseudomaculata*, and *Rhodnius nasutus* were detected; details not shown) and do not investigate correlates of ecotope infestation (for *T. brasiliensis*, such analyses are provided in ref. [21]). Rather, we ask what are the sensitivity and specificity of vector searches, what covariates may induce vector-detection heterogeneity, and how sampling-process uncertainty may affect infestation estimates. In short, this report is an attempt at shedding light on the process of vector detection, and consequently emphasizes practical issues critical to entomological surveillance [16].

We analyzed our detection/non-detection records in two steps. First, we used simple descriptive statistics, considering the results of each vector-search round separately and those of all rounds combined (Tables 1–3). Importantly, these analyses ignore any possible detection errors; this mimics standard practice and yields the naïve ‘infestation indices’ recommended by the World Health Organization [3] – which are used, as far as we are aware, in all Chagas disease control programs. The naïve infestation index is simply II_naïve_  =  *x*/*n*, where *x* is the number of infested sampling units (here, ecotopes with ≥1 detection of vectors or their traces) and *n* is the number of units sampled [3]; for example, with *x* = 50 and *n* = 100, II_naïve_ = 50/100 = 0.50 (or 50%). Although this is routinely interpreted as the proportion (or percent) of sampling units that were infested, we emphasize that it is, in reality, the proportion of sampling units where evidence of infestation was *detected*, usually after a single search. Both quantities would only be equal if evidence of infestation were ascertained without error; they will differ, for example, whenever the sensitivity of the method used to detect infestation is *p*<1.0. For *p* = 0.75, an adjusted estimator of infestation would be II_adjusted_ = *x*/(*n*×*p*) = 50/(100×0.75) ≈0.67. Hence, II_naïve_ will be biased low whenever *p*<1.0, which is probably always [7], [8].

**Table 3:** Observed infestation by Chagas disease vectors in 414 man-made ecotopes of the lower Jaguaribe valley in northeastern Brazil: naïve infestation indices and number of vectors collected.

Locality	Observed infestation		II_naïve_ (%)	Vectors	Density	Crowding
	Yes	No				
Russas	10	199	4.79	14	0.07	1.40
Jaguaruana	87	118	42.44	634	3.09	7.29
Total	97	317	23.43	648	1.57	6.68

II_naïve_, observed infestation index; Vectors, number of triatomine bugs collected in each locality; Density, mean number of vectors across all ecotopes sampled; Crowding, mean number of vectors across ecotopes in which at least one vector was detected; differences between localities were highly significant: observed infestation, Fisher's exact test, *P*<0.0001; observed vector abundance, Wilcoxon rank-sum test, χ^2^ = 27.25, d.f. = 1, *P*<0.0001.

In the second phase of our analyses, we adopt the ‘multiple detection-state’ modeling framework of Miller et al. [9] to explicitly account for possible false-negative (detection failures) and false-positive results (misidentified fecal streaks). We focus on estimating (i) the sensitivity of active vector searches by trained staff (denoted *p*_11_); (ii) the effects of a suite of selected covariates on *p*_11_; and (iii) the probability that an ecotope is incorrectly classified as infested when it is not (*p*_10_, possibly induced by misidentification of fecal streaks) and its complement, 1 – *p*_10_, which estimates vector-search specificity (denoted *s*). Our covariates on *p*_11_ reflect a series of hypotheses about what might affect vector-search sensitivity; after preliminary analyses and prior results from a Jaguaruana data subset (see ref. [21]), we considered three major possibilities:

- Heterogeneity induced by features of our *sampling scheme*, with the sensitivity of vector searches in each ecotope hypothesized to vary (a) among vector-search rounds, with higher sensitivity in the first round (covariate “Search 1”, coded 1 for the result of the first round and 0 otherwise) (see ref. [21]), and/or (b) depending on whether or not, during a given search round, detections had occurred in other ecotopes within the same dwelling compound, which could possibly affect the awareness of field staff as regards vector presence (covariate “SDEc”; for each ecotope and search round, “SDEc” = 1 if one or more detections had occurred in other, same-dwelling ecotopes and 0 otherwise);
- Heterogeneity induced by *differences in vector density*, with sensitivity hypothesized to be higher in more heavily-infested ecotopes. We used the number of bugs collected in each individual ecotope after the third search round as our measure of vector density (covariate “Number of bugs”); the data were standardized to mean 0 and standard deviation (SD) 1 for analysis; and
- Heterogeneity induced by *ecotope characteristics*, with infestation easier/harder to detect in particular ecotope types; we defined the following classes (each coded 1/0): “House”, “Storeroom”, “Henhouse”, “Goat/sheep corral”, “Cattle corral”, “Pigsty”, “Brick pile”, “Tile pile”, and “Woodpile” (Table 1). We also tested whether broader classes (“Building”, “Animal enclosure”, and “Pile”) could explain the data more parsimoniously.

We evaluated these covariates on *p*_11_ as additive terms using the logit link function [9], and used the second-order version of Akaike's information criterion (AICc, with *n* = 414 ecotopes) to rank the models and assess the relative support for each model, given the data [6]–[9], [22]. We fitted 44 models, including a ‘null’ model estimating only intercepts; after preliminary analyses, all models except the ‘null’ included the “Number of bugs” covariate, which clearly improved AICc scores. Models with non-zero Akaike weights (*w*_i_) are presented in Table 4, and the full model set in Table S1.

**Table 4:** The subset of multiple detection-state models with non-zero Akaike weights; models are ranked by their AICc scores.

Detection covariates*	AICc	ΔAICc	*w*_i_	Likelihood	*k*	−2log 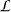
NB+S1+SDEc+BP+WP+Ho+SR+GC	912.41	0.00	0.2836	1.0000	12	887.63
NB+S1+SDEc+BP+WP+Ho+SR+GC+HH	912.76	0.35	0.2381	0.8395	13	885.85
NB+S1+SDEc+BP+WP+Ho+GC+HH	913.47	1.06	0.1669	0.5886	12	888.69
NB+S1+SDEc+BP+WP+Ho+HH	914.35	1.94	0.1075	0.3791	11	891.69
NB+S1+SDEc+BP+WP+Ho+GC	914.57	2.16	0.0963	0.3396	11	891.91
NB+S1+SDEc+BP+WP+Ho+SR+HH	915.30	2.89	0.0669	0.2357	12	890.52
NB+S1+SDEc+BP+WP+Ho+SR	918.42	6.01	0.0140	0.0495	11	895.76
NB+S1+SDEc+BP+WP+Ho	918.51	6.10	0.0134	0.0474	10	897.96
NB+S1+SDEc+BP+WP+Ho+OS	920.61	8.20	0.0047	0.0166	11	897.95
NB+S1+SDEc+BP+WP+Ho+AE	920.62	8.21	0.0047	0.0165	11	897.96
NB+S1+SDEc+BP+WP	922.59	10.18	0.0017	0.0062	9	904.14
NB+S1+SDEc+BP+TP+WP	924.49	12.08	0.0007	0.0024	10	903.94
NB+S1+SDEc+BP+WP+PS	924.61	12.20	0.0006	0.0022	10	904.06
NB+S1+BP+WP+Ho	925.10	12.69	0.0005	0.0018	9	906.65
NB+SDEc+BP+WP+Ho	927.09	14.68	0.0002	0.0006	9	908.64
NB+S1+SDEc+WP	929.56	17.15	0.0001	0.0002	8	913.20
NB+S1+SDEc+TP+WP	931.61	19.20	0.0001	0.0001	9	913.16

*The probability of site-occupancy (or overall infestation prevalence, Ψ) was held constant in all models. Detection parameters include *p*_11_ (probability of detecting infestation in an infested ecotope, or vector-search sensitivity); *p*_10_ (probability of misclassifying a non-infested ecotope as infested); and *b* (probability that a detection is classified as ‘certain’ in an infested ecotope where at least one detection occurred). Each detection parameter was allowed to have a distinct intercept, whereas all parameters had a common slope, as estimated for *p*_11_, for each covariate (see text and Table 5). Detection covariates include: NB, “Number of bugs”; S1, “Search 1”; “SDEc”, detection in same-dwelling ecotopes; BP, “Brick pile”; WP, “Woodpile”; TP, “Tile pile”; Ho, “House”; PS, “Pigsty”; AE, “Animal enclosure”; SR, “Storeroom”; GC, “Goat/sheep corral”; HH, “Henhouse”. See text for the definitions and values of covariates.

AICc, sample size-corrected Akaike's information criterion (or second-order AIC); ΔAICc, difference in AICc between each model and the lowest-AICc (top-ranking) model; *w*_i_, Akaike model weight; Likelihood, likelihood of each model, given the data (or relative strength of evidence for each model); *k*, number of estimable parameters; −2log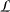, twice the negative log-likelihood of each model. See ref. [22] for formulae and details on AICc and related metrics.

Apart from sensitivity (*p*_11_) and covariate effects, our models also estimate (i) a site-occupancy parameter (denoted Ψ) that expresses the mean probability that an ecotope is infested (or, equivalently, overall infestation prevalence); (ii) the probability of false-positive detections, *p*_10_; and (iii) the probability that a detection is classified as ‘certain’, given the ecotope is infested and a detection occurred (denoted *b*) [9]. For simplicity, Ψ was held constant in our current models, which as mentioned above focus on the vector-detection process and especially on the sensitivity of active vector searches (*p*_11_). Covariate effects were allowed to modify *p*_11_, *p*_10_ and *b*, so that detection parameters had different intercepts but common slopes; we tested alternative parameterizations, either with *p*_10_ fixed at zero (i.e., assuming no false-positive results) or with *p*_10_ and *b* varying only with observed bug density and sampling-scheme covariates (“Search 1” and “SDEc”), but the models had larger AICc scores (details not shown). We calculated model-averaged estimates of Ψ and covariate effects on *p*_11_, with unconditional standard errors (SEs), using equations 4.1 and 4.9 in ref. [22]. For detection parameters *p*_11_, *p*_10_, and *b* (and their SEs), we calculated model-weighted averages of individual results (i.e., model-predicted values and SEs for each individual ecotope and search round, weighted by each model's *w*_i_) and provide summary statistics (see Table S2). For consistency with our AIC-based approach, we present parameter and covariate-effect estimates with approximate 85% confidence intervals (CIs) (see ref. [23]), although we also comment on the more conventional 95%CIs in some instances. Models were fit via maximum likelihood as implemented in Presence 6.4 [24].

We finally compared the results of naïve and model-based analyses in the epidemiologically- and operationally-relevant terms of (i) estimates of infestation prevalence (II_naïve_ *vs*. model-averaged Ψ) and (ii) estimates of the number of infestation foci that likely went undetected during standard, active vector searches.

## Results

### Descriptive results

Naïve infestation indices for each vector-search round and all rounds combined are presented in Table 1 (see raw data in Dataset S1). Overall, more detections occurred during the first than during the second and third vector-search rounds (see also [21]); a similar trend was apparent when considering ‘certain’ detections only. Importantly, naïve infestation indices were higher in almost all ecotope types when the results of the three vector-search rounds were combined than when considering each single round in isolation (Table 1). Over all ecotope types, combined-search naïve infestation indices were from 1.24 to 2.06 times higher than single-search indices for all detection data, and from 1.30 to 1.98 times higher than single-search indices for ‘certain’ detection data.

Table 2 summarizes ‘detection histories’ for the 414 ecotopes surveyed. Evidence of infestation was detected at least once in 19 ecotopes where the first vector search had yielded no detections. In 21 ecotopes only the first vector search yielded evidence of infestation, with ‘certain’ detections (history “200”) in 16 ecotopes. Evidence of vector presence was consistently found across all search rounds in only 30 of the 97 ecotopes where such evidence was found at least once; ‘certain’ detections consistently occurred in 22 of those ecotopes. Thus, many observed infestation foci went undetected during single vector-search rounds: at least 19 foci in the first, 36 in the second, and 50 in the third round. Considering only the 87 ‘certain’ observed foci, 20 were missed in the first, 42 in the second, and 43 in the third search round. We finally note that observed infestation was markedly different in our two study localities, with triatomine bug foci apparently more common and denser in Jaguaruana than in Russas (Table 3).

### Modeling

**Figure 1:**
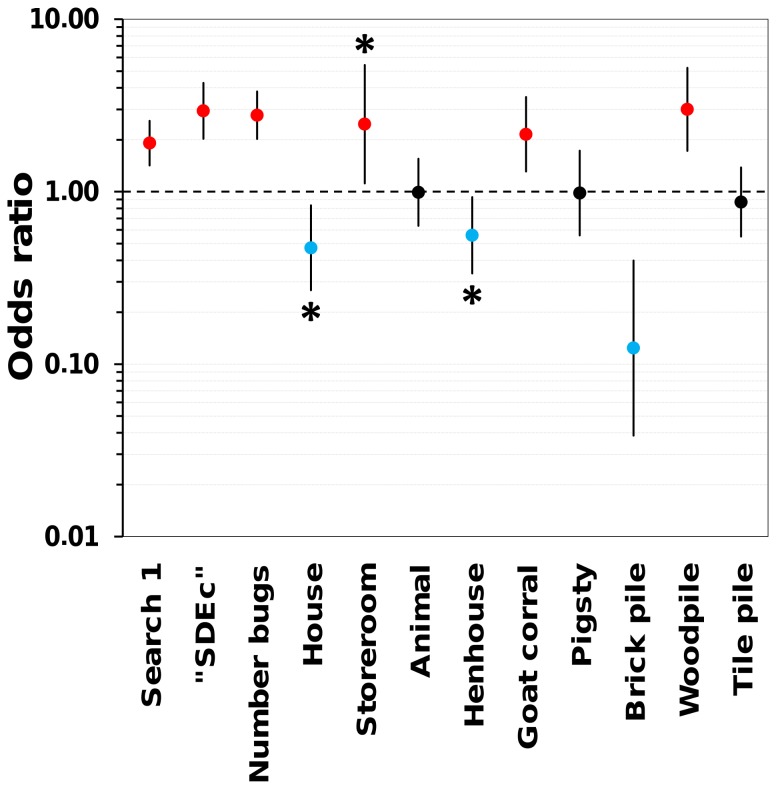
Effects of covariates on the sensitivity of active Chagas disease vector searches in the lower Jaguaribe valley, Ceará, Brazil: model-averaged odds ratios (ORs) with approximate 85% confidence intervals (CIs) based on unconditional standard errors. The effect of a covariate is considered indistinguishable from zero when the CI crosses the dashed line at OR = 1.0 (black circles), positive if all values are >1.0 (red circles), and negative if all values are <1.0 (blue circles). Asterisks highlight covariates whose 95%CI overlaps 1.0. “SDEc” indexes, for each ecotope and vector-search round, whether detections occurred in other, same-dwelling ecotopes; see main text for further details. For each covariate effect (*β*_i_), the OR is estimated as OR =  exp(*β*_i_).

**Figure 2:**
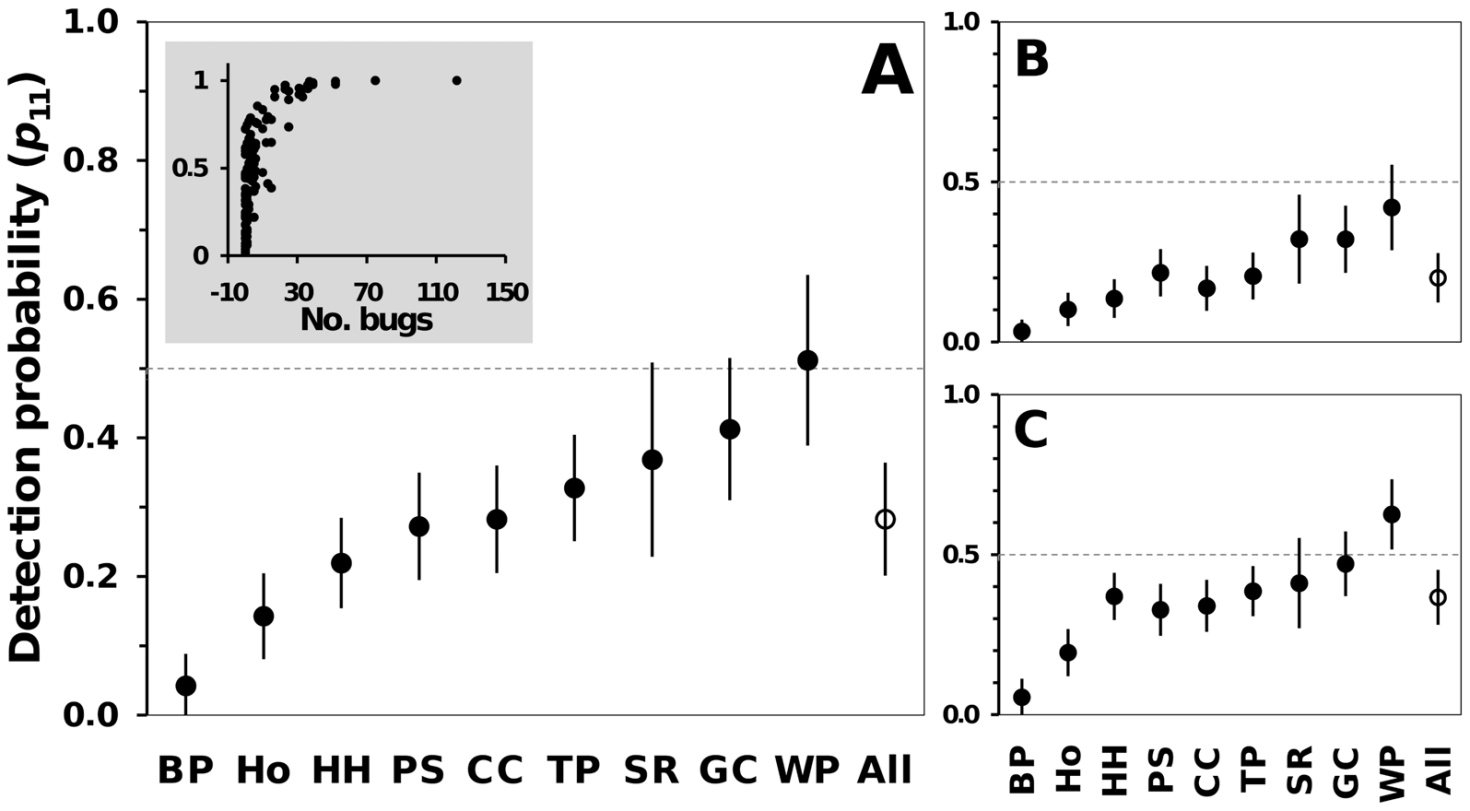
Model-weighted average estimates of Chagas disease vector-search sensitivity (*p*_11_) for different ecotope types. The means of model-averaged ecotope- and vector-search round-specific values are shown, with approximate 85% confidence intervals; in each panel, the mean vector-search sensitivity over all ecotope types (labeled “All”) is represented by an empty circle, and 50% sensitivity is highlighted by dashed lines. **A**, estimates from the complete dataset, with ecotopes ranked by mean vector-search sensitivity; the inset shows the relationship between model-predicted sensitivity and observed vector density; **B**, estimates for the lightly-infested locality of Russas; **C**, estimates for the heavily-infested locality of Jaguaruana. Ecotopes: BP, brick pile; Ho, house; HH, henhouse; PS, pigsty; SR, storeroom; GC, goat/sheep corral; CC, cattle corral; TP, tile pile; WP, woodpile. See Table S2 for further details.

Site-occupancy models with non-zero Akaike weights (Σ*w*_i_ = 1.0) are presented in Table 4. Model-averaged, adjusted estimates of covariate effects on vector-search sensitivity (*p*_11_), along with their unconditional SEs and 85%CIs, are presented in Table 5; the associated odds ratios are shown in Figure 1. The sensitivity of active vector searches was higher in ecotopes harboring denser bug colonies; the odds of vector detection were 2.77 (85%CI 2.02–3.80) times higher for each unit increase in the standardized number of bugs caught in a given ecotope (1 SD increase ≈9 bugs) (Fig. 1 and inset in Fig. 2A). Vectors were also easier to detect in peridomestic woodpiles (odds ratio 3.00, 85%CI 1.72–5.23), in goat/sheep corrals (2.15, 85%CI 1.31–3.54), and during the first search round (1.91, 85%CI 1.42–2.57); the positive effect of storerooms (odds ratio 2.46) was associated with larger uncertainty (85%CI 1.12–5.42, with the 95%CI encompassing 1) (Fig. 1). Vector-search sensitivity was somewhat lower in houses (odds ratio 0.47, 85%CI 0.27–0.83; upper limit of the 95%CI = 1.02) and henhouses (0.56, 85%CI 0.34–0.93; upper limit of the 95%CI = 1.12), and substantially lower in brick piles, albeit uncertainty about this latter estimate was large (odds ratio 0.12, 85%CI 0.04–0.40) (Fig. 1). Other covariates, including those grouping buildings, animal enclosures and piles, had no discernible effects on *p*_11_ (Tables 5 and S1, Fig. 1).

**Table 5:** Model-averaged, adjusted slope coefficient estimates for detection covariates appearing in the subset of models with non-zero Akaike weights (see Table 4).

Group	Covariate	Slope coefficient	SE	85%CI	
				Lower	Upper
Sampling scheme	Search 1	**0.65**	0.21	0.35	0.95
	“SDEc”	**1.08**	0.26	0.71	1.45
Observed vector density	Number of bugs	**1.02**	0.22	0.70	1.34
Buildings	House	−0.75*	0.39	−1.32	−0.18
	Storeroom	0.90*	0.55	0.11	1.69
Animal enclosures	Animal enclosure	−0.01	0.31	−0.46	0.44
	Henhouse	−0.58*	0.35	−1.09	−0.07
	Goat/sheep corral	**0.77**	0.35	0.27	1.26
	Pigsty	−0.02	0.39	−0.58	0.55
Piles	Brick pile	**−2.09**	0.81	−3.26	−0.92
	Woodpile	**1.10**	0.39	0.54	1.65
	Tile pile	−0.14	0.32	−0.60	0.32

Slope coefficient, model-averaged slope coefficient point estimate; SE, unconditional standard error; Lower and Upper, lower and upper limits of the approximate 85% confidence interval (CI). “SDEc”, detection in same-dwelling ecotopes. Coefficients highlighted in **bold** typeface have 85%CIs not overlapping zero; asterisks (*) indicate estimates whose 95%CI overlaps zero.

With our parameterization, models including detection covariates estimate ecotope-specific values for *p*_11_, *p*_10_, and *b* [9]; we therefore provide summary statistics of model-averaged estimates for each parameter and its variation. Figure 2 shows model-averaged *p*_11_ estimates for different ecotopes; sensitivity was overall low (mean across ecotopes and vector-search rounds, *p*_11_≈0.283±0.057; median  = 0.231, inter-quartile range 0.123–0.384), and particularly so in brick piles (mean *p*_11_≈0.042±0.032) and houses (mean *p*_11_≈0.143±0.043). Overall, sensitivity was lower in the lightly-infested (mean *p*_11-Russas_≈0.200±0.054; Fig. 2B) than in the heavily-infested locality (mean *p*_11-Jaguaruana_≈0.367±0.060; Fig. 2C). Sensitivity was estimated at *p*_11_≈1.00 for a single tile pile where 122 triatomine bugs were collected after the third search round. See Table S2 for further details about *p*_11_ values.

An ecotope can be incorrectly classified as infested, with probability *p*_10_, when infestation status is determined based on the detection of fecal streaks. Our models suggest that this event was, on average, very unlikely: the mean of model-averaged values across ecotopes and vector-search rounds was *p*_10_≈0.011±0.008 (median  = 0.0015, inter-quartile range 0.0007–0.0030), reaching high values (>0.90) in the few ecotopes where *p*_11_ was also very high. This reflects the fact that the detection of *only* fecal streaks in ecotopes where sensitivity is close to 100% almost surely represents a false-positive result. Hence, with a few exceptions, vector-search specificity (*s* = 1 – *p*_10_) was reassuringly high, with a mean value of ∼0.989. The probability that a detection was classified as ‘certain’, given the ecotope was infested and at least one detection occurred, was moderately high (mean across ecotopes and vector-search rounds, *b*≈0.637±0.073) and varied from 0.116 in 19 brick piles to ∼1.0 in the tile pile where *p*_11_ was also ∼1.0.

**Figure 3:**
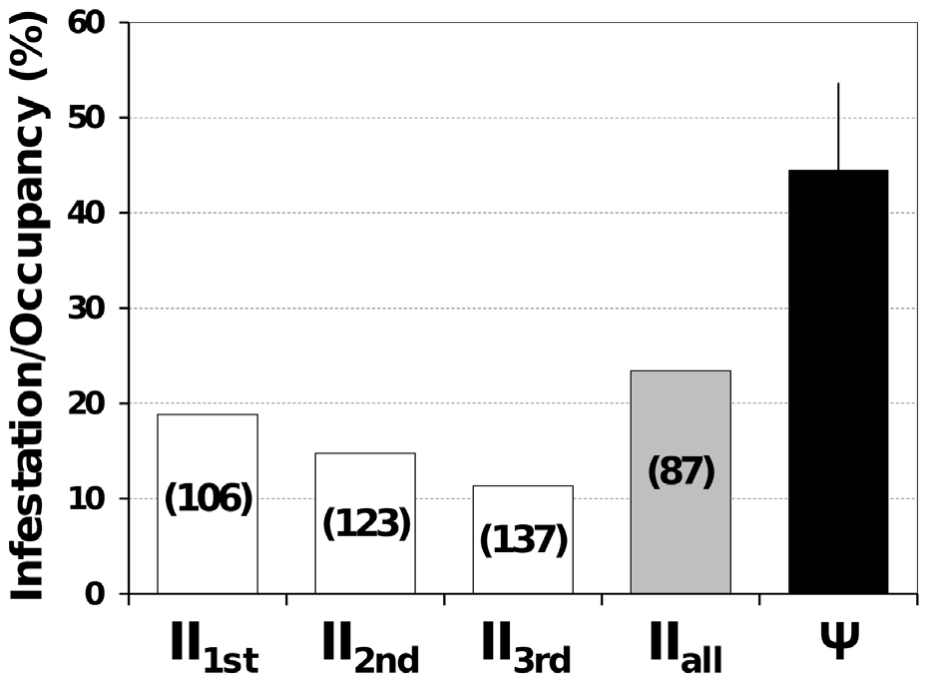
Infestation by synanthropic Triatominae in 414 man-made ecotopes of the Jaguaribe valley, Ceará, Brazil. II, naïve infestation index from results of single vector-search rounds (II_1st_ to II_3rd_) and all rounds combined (II_all_); Ψ, model-averaged site-occupancy estimate (error bar, 85% confidence interval). The estimated numbers of infestation foci that went undetected during single vector-search rounds and all rounds combined are shown inside the corresponding bars.

Model-averaged infestation prevalence (or mean ecotope-occupancy rate) was estimated as Ψ_average_≈0.445 (unconditional SE = 0.064; 85%CI 0.353–0.537); this estimate is nearly twice as high as the naïve infestation index calculated with the combined results of three vector-search rounds: II_naïve_ = 97/414 = 0.234 (Fig. 3). Our model-based site-occupancy estimate suggests that the number of infested ecotopes was *x*′ = Ψ_average_ × *n* = 0.445×414≈184; therefore, and despite triplicate search effort, as many as ∼87 infestation foci most likely went undetected during active vector searches. Considering the results of single vector-search rounds separately (which is standard practice in vector surveillance and research), we estimate that about 106, 123, and 137 infestation foci went undetected during the first, second, and third search rounds, respectively (Fig. 3). Importantly, our analyses suggest that vector-search sensitivity was especially poor in the more lightly-infested locality of Russas (Fig. 2), where observed infestation prevalence (II_naïve-Russas_ = 0.048; Table 3) was therefore likely to be particularly biased low.

## Discussion

**Figure 4:**
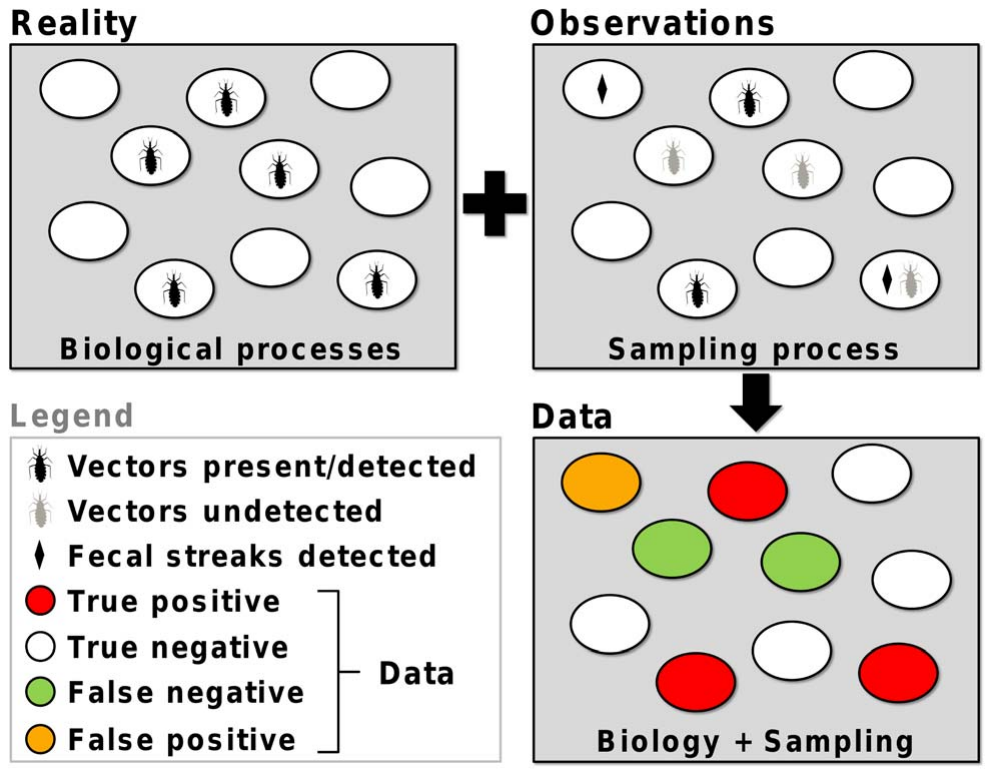
Full reality is only partially observed. Imagine a survey in which 10 discrete ecotopes, five of which are infested (“Reality”), are searched for vectors or their traces. During the survey (“Observations”), the vectors went undetected in two infested ecotopes; non-vector fecal streaks were misidentified as vector feces in one ecotope; vectors were detected in two infested ecotopes; vector fecal streaks were correctly identified in a third infested ecotope where no vectors were detected; and no detections occurred in four non-infested ecotopes. The resulting dataset (“Data”) is therefore a biased representation of reality – it contains one false-positive detection and two false-negative results, along with three true-positive and four true-negative results.

In spite of obvious implications for vector-borne disease research and control-surveillance, little is known about the uncertainties associated with sampling disease vectors under realistic field survey conditions [5], [6], [14]–[17], [25]. Somewhat surprisingly, the conventional approach to sampling-process uncertainty has been to formally ignore it. Thus, the most authoritative global public health agencies recommend the use of infestation indices that rely on the implicit assumption that vectors are detected without error (e.g., [2], [3]). As a result, observed detection/non-detection data are usually treated as if they were *true* presence/absence data, yet they are not: in virtually any real-life scenario, true vector presence/absence is only partially observed (Fig. 4). Detection errors can plague not only overall infestation measures, but also other commonly-used naïve ‘entomological indicators’, including ‘intradomiciliary’ and ‘peridomestic’ infestation, ‘colonization’, ‘density’, ‘dispersion’, or ‘natural infection’ indices (see Box 2 of ref. [3]). The definitions of these indicators should stress that what we can really measure is whether vectors or pathogens were present and detected – and that, even then, some detections may be spurious [5]–[9], [26].

Put another way, we must acknowledge that, in most of our datasets, heterogeneity induced by the *biological* processes governing vector (or pathogen) occurrence is confounded with heterogeneity induced by the *sampling* process governing vector (or pathogen) detection (Fig. 4). Here we have shown that this need not be so; relatively straightforward approaches to disentangling biological- and sampling-process variation are readily available, allowing for detailed investigation of the determinants of vector/pathogen occurrence and the determinants of vector/pathogen detection. This can foster our understanding of infectious disease ecology and may transform our view of how control-surveillance systems actually perform [16], [25], [26].

In this report we focused on quantitatively investigating the process of Chagas disease vector detection in man-made environments while realistically considering false-positive and false-negative detection errors; given this focus, we largely ignored biological issues [21]. Hierarchical models, however, address occurrence and detection simultaneously; here, we set Ψ to be constant for clarity and simplicity, and because of our specific research question. We also note that adding covariate structure to the occupancy part of our top-performing model (Table 4) increased AICc scores: the most parsimonious such model, which included just one covariate on Ψ (“Goat/sheep corral”; *β* = 0.688, SE = 0.551), performed no better than our second-ranking model (Table 4) and estimated detection-covariate effects consistent in sign and size with those presented in Table 5 (details not shown). That constant-Ψ models tend to fit well is most likely related to our pooling of species-specific data for analysis: what favors occupancy by one species may have a negative effect on another. Given our focal aim and these considerations, we present and discuss the results of our simpler models with constant occupancy.

Our analyses show that, after adjusting for variation induced by operational details (see below), the sensitivity of the standard Chagas disease vector-surveillance method (active searches by trained staff) is overall low and can vary widely depending on vector density and ecotope traits (Figs. 1 and 2). Using this information, we evaluated how sampling errors might affect infestation estimates, and showed that naïve indices can be badly biased low, with many infestation foci going undetected (Fig. 3). Finally, we provided estimates of other important sampling-process parameters [9] including the probability of false-positive detections, which was reassuringly low, and the probability of detections being classified as ‘certain’ in ecotopes where vectors were present and a detection had occurred, which was fairly high but variable.

One potential caveat of our study is that we treated detection/non-detection events as independent; it seems likely, however, that the detection/non-detection of vectors or their traces in one ecotope may affect the probability of detection/non-detection in nearby ecotopes. For example, detection in one ecotope might increase awareness of the vector-search team regarding the possibility of vector presence in other ecotopes within the same dwelling compound. We modeled this possible source of heterogeneity with our “SDEc” covariate, which had a positive effect (Table 5, Fig. 1). Of more relevance to our aims, inclusion of the “SDEc” covariate in the models allowed us to derive adjusted effect-size estimates for the covariates of practical interest, such as those describing observed bug density or indexing ecotope types. We acknowledge, however, that there could be further spatial dependencies (e.g., among ecotopes in neighboring dwelling compounds) that the “SDEc” covariate does not capture. Another potential source of variation we wanted to adjust for was variation among search rounds. A previous analysis of data from Jaguaruana, focusing on *T. brasiliensis* site-occupancy, suggested that sensitivity was higher in the first search round (see ref. [21]). This was confirmed in the present analyses (Table 5); removal of the “Search 1” covariate from our top-ranking model resulted in a ΔAICc≈8.0 (details not shown). As with “SDEc”, effect-size estimates for covariates of more practical interest (Table 5) adjust for this variation. Apart from independence among sites and search rounds, our models also assume population closure (no local extinction or colonization) over the survey period, which the short sampling time-frame and the low vagility of triatomines virtually ensured [21].

A further caveat refers to the use of the number of bugs collected in each ecotope as a proxy for vector density. To be consistent with the conceptual framework of imperfect detection, we have to acknowledge that we did not know how many individual vectors went undetected in each ecotope, including ecotopes with zero detections. The “Number of bugs” covariate has therefore to be regarded as a rough approximation to differences in vector density among ecotopes; as expected, the effect of increasing observed density on detection probability was obviously positive and moderately large (Table 5, Fig. 1).

We recall that observed vector density and infestation prevalence were both higher in Jaguaruana than in Russas (see Table 3). Our study localities hence mirror two common scenarios in Chagas disease vector control: (a) a typical pre-control (or control-breakdown) scenario in Jaguaruana, with many, relatively large infestation foci, and (b) a typical post-control scenario in Russas, with just a few, small vector foci (see, e.g., [17], [27]). By comparing locality-specific model predictions, we were therefore able to approximate how such scenarios may induce vector-detection heterogeneity; our results show that differences can be important, with vector-detection sensitivity consistently lower in Russas than in Jaguaruana (Fig. 2). This observation suggests that naïve infestation indices may be especially deceitful in lightly-infested localities such as those undergoing ‘successful’ vector control (see also [17], [27]). This has obvious implications for control-surveillance programs: larger negative bias in post-control infestation indices may lead to overly optimistic views of vector control performance and disease transmission risk [27]–[29].

We however caution that our quantitative results (i.e., parameter and effect-size numerical estimates) cannot be extrapolated to unsampled areas or ecotopes – if anything else because we did not sample probabilistically from any known study universe of localities or ecotopes. We nonetheless believe that our results have important implications in that negatively-biased infestation indices and the sampling-process uncertainties underlying such bias are, in all likelihood, general features of disease-vector surveys – in Chagas disease [5], [6], [16], [17], [21], [25], [27]–[29] and in other systems including arboviral diseases such as dengue or chikungunya [30], flea-transmitted plague [31], or sandfly-transmitted leishmaniasis (FA-F, unpublished). Regarding Chagas disease, the negative effect of lighter infestation on sensitivity, with bias getting worse as vector density declines, is almost certainly a widespread issue [14]–[17], [27], [29]. In contrast, ecotope-type effects are unlikely to be general: our finding of lower sensitivity in brick piles and houses, and higher sensitivity in woodpiles and goat/sheep corrals, probably reflects, at least partially, micro-habitat preferences of the most common local triatomine species, *T. brasiliensis* [21].

At any rate, knowing that such sampling-process heterogeneities exist and can be substantial is obviously very relevant. In vector ecology research, ignoring this variation could lead to wrong conclusions about drivers of site-occupancy; for example, ecotopes in which sensitivity is lower could be incorrectly classified as low-quality habitat. In vector control-surveillance, this knowledge might be used to target vector-search effort according to operational objectives and ecotope types; for example, when a survey aims at determining infestation at the *dwelling* level (which is the case in most programs), vector searches could start in ecotopes where sensitivity is highest. This would probably save search effort, but would also require periodically running pilot surveys to estimate vector-search sensitivity and how it varies in operationally- (e.g., municipalities) or ecologically-relevant units (our results, for example, likely apply over the middle-lower Jaguaribe valley and perhaps in other similar sedimentary Caatinga lowlands).

Finally, we again emphasize that, although here we were specifically interested in studying the detection of triatomine bugs (or their traces) in man-made ecotopes, the underpinnings of our approach apply to the detection/non-detection of any organism (or its traces) in any discrete sampling unit [9]. Analogous situations arise, for example, when investigating the patterns of ‘occupancy’ of individual organisms (e.g., persons or vectors) by infectious disease agents for which detection (diagnostic) methods can yield false-positive and false-negative results [9], [26]. Thus, the approach finds application whenever some diagnoses can be classified as ‘certain’ (e.g., unambiguously identifying a parasite in a microscope slide) and some as ‘uncertain’ – e.g., detecting anti-parasite antibodies (possibly with cross-reactions) or parasite DNA (with uncertainty about, say, parasite viability or the possibility of sample contamination). In these and similar situations, multiple detection-state and multiple detection-method models can be used to reduce bias in parameter estimates [9], [26].

### Conclusions and outlook

We have presented a detailed investigation of major sources of detection heterogeneity in Chagas disease vector surveys. To our knowledge, this is the first attempt at quantifying vector sampling uncertainty when survey methods can yield spurious detections and non-detections. Our results are far from encouraging: they suggest that discounting sampling-process uncertainty, and particularly false-negative results, can lead to serious, overoptimistic misrepresentations of both disease transmission risk and vector control performance. Reliable measures of disease vector (or pathogen) presence/absence are essential for disease prevention; while it is unfortunate that available triatomine-detection tools perform poorly, with sensitivity typically below 50%, ignoring this critical problem will not solve it. Instead, we must develop a sound understanding of how the vector-detection process works and incorporate the associated uncertainties into our operational indicators. The approach we used here can help do so. We expect that, sometime in the near future, the crucial issue of sampling-process uncertainty will be widely acknowledged, and formally accounted for, in routine-surveillance systems. Otherwise, many human beings will continue to suffer vector presence and disease transmission while researchers, control managers and international-agency officials, misled by imperfect data, celebrate public health ‘achievements’ that may well glister but are not gold [28], [29].

## Supporting Information

Dataset S1 Raw data: vector-search results and covariate values.
(XLSX)

Table S1 The complete set of multiple detection-state site-occupancy models. AICc, Akaike information criterion corrected for sample size; ΔAICc, difference in AICc between each model and the lowest-AICc (top-ranking) model; *w*_i_, Akaike model weight; Likelihood, likelihood of each model, given the data (or relative strength of evidence for each model); *k*, number of model parameters; Deviance, –2log-likelihood of each model. See main text for the definitions and values of covariates.
(XLSX)

Table S2 Summary statistics for model-averaged vector-search sensitivity estimates: means, medians, quartiles and ranges of ecotope- and vector-search round-specific *p*_11_ values; mean values for standard errors (SE), and upper/lower limits of the approximate 85% confidence intervals (CI).
(XLSX)
